# Social inequalities in cancer in Germany: a call to action

**DOI:** 10.1007/s00432-026-06438-4

**Published:** 2026-03-10

**Authors:** Nora Tabea Sibert, Maike Wellbrock, Ahmed Bedir, Nina Grundmann, Markus Herrmann, Jens Hoebel, Lina Jansen, Vera Popova, Lars Schwettmann, Volker Arndt, Tilman Brand, Anne-Christin Gude, Connie Hoe, Joachim Hübner, Milena Lückemeyer, Ute Mons, Susanne Oksbjerg Dalton, Sven Voigtländer, Friederike Erdmann

**Affiliations:** 1 Oncological Health Services Research, Clinic for Gynaecology and Obstetrics, University Clinic Düsseldorf, Düsseldorf, Germany; 2 Department of Health Services Research, German Cancer Society, Berlin, Germany; 3 Centre for Integrated Oncology Aachen, Bonn, Cologne, Düsseldorf (CIO ABCD), Düsseldorf, Germany; 4 Centre for Health and Society, University Clinic Düsseldorf, Düsseldorf, Germany; 5 Research Group Aetiology and Inequalities in Childhood Cancer, Division of Childhood Cancer Epidemiology, Institute of Medical Biostatistics, Epidemiology and Informatics (IMBEI), University Medical Center Mainz, Mainz, Germany; 6 Department of Prevention and Evaluation, Leibniz Institute for Prevention Research and Epidemiology (BIPS), Bremen, Germany; 7 Cancer Registry Saxony-Anhalt, Magdeburg, Germany; 8 University Clinic of Radiation Oncology, Medical Faculty of Otto-Von-Guericke-University Magdeburg, Magdeburg, Germany; 9 Bavarian Cancer Registry, Bavarian Health and Food Safety Authority, Nuremberg, Germany; 10 Faculty of Medicine, Institute for Medical and Data Ethics, Heidelberg University, Heidelberg, Germany; 11 National Center for Tumor Diseases (NCT), NCT Heidelberg, a Partnership Between DKFZ and Heidelberg University Hospital, Germany, German Cancer Research Center (DKFZ), Heidelberg, Germany; 12 Division of Social Determinants of Health, Department of Epidemiology and Health Monitoring, Robert Koch Institute, Berlin, Germany; 13 Epidemiological Cancer Registry Baden-Württemberg, German Cancer Research Center (DKFZ), Heidelberg, Germany; 14 Bavarian Cancer Registry, Bavarian Health and Food Safety Authority, Augsburg, Germany; 15 Department of Health Services Research, Faculty VI - School of Medicine and Health Sciences, Carl Von Ossietzky Universität Oldenburg, Oldenburg, Germany; 16 Unit of Cancer Survivorship, German Cancer Research Center (DKFZ), Heidelberg, Germany; 17 Institute for Diversity Medicine, Ruhr-University Bochum, Bochum, Germany; 18 Division of Policy and Implementation Research for Cancer Prevention, DKFZ Hector Cancer Institute at the University Medical Center Mannheim, Germany, German Cancer Research Center (DKFZ), Heidelberg, Germany; 19 Agency for Clinical Cancer Data of Lower Saxony, Oldenburg, Germany; 20 Division of Primary Cancer Prevention, German Cancer Research Center (DKFZ), Heidelberg, Germany; 21 Medical Faculty Mannheim, Heidelberg University, Mannheim, Germany; 22 Cancer Survivorship, Danish Cancer Institute, Copenhagen, Denmark; 23 Department of Clinical Oncology and Palliative Care, Danish Research Center for Equality in Cancer (COMPAS), Zealand University Hospital, Næstved, Denmark; 24 Faculty of Health Sciences, Institute of Clinical Medicine, University of Copenhagen, Copenhagen, Denmark

**Keywords:** Health equity, Social inequalities, Social epidemiology

## Abstract

**Background:**

Social inequalities in cancer constitute a major public health challenge. A lower socioeconomic position (SEP) is consistently associated with higher exposure to cancer risk factors, lower participation in screening, more advanced stage at diagnosis, poorer survival, and adverse survivorship outcomes. In Germany, these inequalities remain insufficiently addressed in research and health policy.

**Methods:**

This paper synthesises evidence and expert perspectives derived from a national workshop organised by the Cancer Epidemiology Working Group of the German Society for Epidemiology (DGEpi) in collaboration with the German Cancer Research Center. More than 30 experts in cancer epidemiology and social inequality research, together with international contributors, reviewed—based on existing conceptual frameworks—the German data landscape, and empirical evidence across the cancer continuum. Structural, methodological, and ethical barriers were identified, and implications for research, policy, and practice were discussed. An international comparison with Denmark was used to contextualise findings.

**Results:**

Available evidence demonstrates pronounced socioeconomic inequalities across nearly all stages of the cancer continuum in Germany, including prevention, screening, incidence, diagnosis, survival, and survivorship. However, major research gaps persist. Key barriers include limited availability of individual-level SEP data, reliance on area-based deprivation indices, restricted data linkage, fragmented healthcare structures, and limited integration of equity considerations into national cancer strategies. International experience shows that comprehensive registries, data linkage, and targeted interventions can reduce inequalities.

**Conclusions:**

Reducing social inequalities in cancer in Germany requires coordinated and evidence-based action. Priorities include improving SEP data availability and linkage, embedding equity objectives into the National Cancer Plan, implementing targeted interventions for vulnerable groups, and strengthening intersectoral collaboration. Ethical and patient perspectives strongly support responsible use of health data to address avoidable inequalities.

## Introduction

Monitoring and tackling social inequalities in health has become a key priority for public health policy worldwide. Health inequalities rooted in social conditions are widely regarded as both unjust and, in principle, avoidable. They arise not only from individual-level characteristics, but also from socially patterned living and working conditions, neighbourhood environments, and institutional arrangements that systematically shape exposure to cancer risk factors and access to resources such as prevention and care (Solar and Irwin 2010; Sarfati 2019). A rapidly growing body of evidence demonstrates social inequalities in cancer, indicating that social determinants are associated with cancer incidence, mortality, and survival not only globally, between countries of varying socioeconomic development, but also within high-income countries (Bray 2014; Dalton et al. 2008). Social inequality in cancer is present when a systematic association exists between the socioeconomic position (SEP) of an individual within a society and cancer-related outcomes such as screening participation, incidence, diagnosis, treatment, or survival (Olsen et al. 2023; Vaccarella et al. 2019).

In high-income countries, where substantial advances in cancer diagnosis and treatment resulted in a steadily growing population of cancer survivors, receiving a cancer diagnosis itself is a potential driver of social inequalities. The adverse social consequences experienced by survivors and their families, including financial hardship, disrupted employment trajectories, and long-term psychosocial burdens, are therefore gaining increasing societal recognition (de Boer et al. 2009; Kirchhoff et al. 2012; Roick et al. 2022; Sibert et al. 2025).

In Germany, evidence indicates that inequalities across the cancer continuum are substantial yet remain insufficiently studied as well as recognised in national health policy. Socioeconomically disadvantaged groups show lower participation in preventive services and face barriers in access to timely and high-quality care (Schülein et al. 2017; Klein et al. 2025). European Union (EU)-wide programmes such as the EU Commission’s “Europe beating cancer plan” are explicitly aiming at “[eradicating] inequalities in access to cancer knowledge, prevention, diagnosis and care” (European Commission 2022). At the same time, systematic research on these inequities has only recently gained momentum. To accelerate progress, the Cancer Epidemiology Working Group of the German Society for Epidemiology (DGEpi), in collaboration with the German Cancer Research Center (DKFZ), convened a workshop on *“Social Inequalities in Cancer: Potential and Approaches for Achieving Greater Equity”* in Heidelberg in December 2024. The workshop brought together 33 epidemiologists and public health scientists from across Germany, complemented by two international experts from Denmark and the UK, to analyse inequalities in the cancer continuum and discuss approaches for reducing them.

In the following, we describe existing conceptual frameworks, review the German evidence and data landscape, discuss ethical aspects, barriers, and opportunities, and compare the German situation with international experiences. We conclude with a call for action and perspectives for future research and policy.

## Conceptualizing social inequalities in cancer

Social inequalities in cancer encompass systematic differences between social groups across the entire cancer continuum, from exposure to risk factors and incidence of cancer to participation in screening programmes, access to diagnosis and treatment, provision of treatment, follow-up, palliative care, survival, and survivorship (Sarfati 2019).

SEP (“socioeconomic position”) is a dynamic multidimensional construct, reflecting both material and social resources, assets and stressors, which are rooted in specific social contexts and, more fundamentally, in an individual’s rank or status within the social hierarchy of a society. SEP comprises different socioeconomic dimensions, which may impact health at different phases across the life course and through different causal pathways. Our conceptualization of social inequalities in cancer is based on levels, social contexts, time, and life course and, thus, helps to describe and explicate specific “causal pathways that involve exposure, susceptibility, and resistance (as both social and biological phenomena)” (Krieger 2008). We use the term “socioeconomic position” (SEP) rather than the more commonly used term “socioeconomic status” (SES) following Krieger et al. (Krieger et al. 1997), who argue that SEP not only encompasses actual resources (such as income and wealth) but also status (which relates to social rank and prestige).

Theoretical frameworks in social epidemiology differ from biomedical and lifestyle models of disease as they go beyond the individual as the sole unit of analysis (Krieger 2019). Typically, these frameworks distinguish the macro level of social inequality from the micro level of the individual, which are linked by the meso level (or several in-between levels) including multiple social contexts or domains (Krieger 2019; Solar and Irwin 2010; Steinkamp 1993). More specifically, social inequality at the macro level is produced by, e.g. economic and political priorities of societies encompassing inequality according to social groups, gender, ethnicity, as well as entitlements related to the welfare state and social security, also defined as SEP (Krieger 2008; Krieger et al. 1997). The macro level shapes the meso level as it gives rise to exposures (resources as well as stressors) in social contexts where people live and work, such as partner relationships, families, peer groups, work, school and other public settings. Comprising the personality and the individuals’ organism, the micro level is the level where the interplay of resources and stressors is experienced (Steinkamp 1993) and where the effects of the upper levels are shaped by differential vulnerability and susceptibility, meaning that identical exposures may have stronger adverse effects in lower SEP groups (Diderichsen et al. 2018). At the micro level, exposures are, thus, biologically incorporated, what Krieger (2019) describes as “embodiment”. Emerging research in human social genomics demonstrates that social stressors can influence gene expression, highlighting the biological embeddedness of social inequalities (Cole 2014). The frameworks also stress the importance of time regarding the temporal distance to exposure, phases of the life course (e.g., in utero, infancy, childhood, and adulthood), and the duration or persistence of exposure, for instance, of living in a specific social group or a specific social context. It is important to add that the micro level can in return affect the SEP as is the case e.g. for cancer survivorship and unemployment (de Boer et al. 2009).

Smoking, as one of the major behavioural risk factors for cancer, provides an excellent example for illustrating this conceptual framework in the context of cancer. The most used measures of SEP are education, income or wealth and occupation. For all indicators, there is established evidence of adverse influence from lower SEP on smoking behaviour: unemployment is associated with a higher risk for smoking (Arcaya et al. 2014), as is lower income (Hiscock et al. 2012) and lower level of education (Uong et al. 2024). Thus, as Hiscock at el. concluded already in 2012: “Smoking prevalence is higher among disadvantaged groups, and disadvantaged smokers may face higher exposure to tobacco's harms.”—especially cancer (Hiscock et al. 2012). The causal mechanisms can be conceptualised across the different levels outlined above. At the micro level, disadvantaged individuals may experience higher exposure to tobacco’s harms due to greater biological and psychosocial vulnerability. Chronic stress, allostatic load, and cumulative disadvantage can amplify the physiological effects of tobacco (Stringhini et al. 2010; McEwen and Gianaros 2011), while limited health literacy may reduce the capacity to adopt cessation strategies. At the meso level, smoking behaviour is reinforced through contextual and relational factors such as work-related exposure, as well as family and social networks that normalise or encourage smoking. For example, a spouse’s unemployment (Arcaya et al. 2014) and parental education (Yáñez et al. 2017) have been identified as predictors of smoking status. At the macro level, structural and commercial determinants influence smoking prevalence: Lower SEP groups are disproportionately targeted by tobacco marketing and face environments that facilitate tobacco use (e.g., density of tobacco retailers in disadvantaged neighbourhoods) (Kong et al. 2022). Legislative measures such as increasing tobacco prices through taxation have been shown to reduce social inequalities in smoking (Hill et al. 2014).

Different mechanisms have been proposed to mediate the effect of SEP across the cancer continuum, including occupational exposures, differences in health literacy, differential exposure to behavioural risk factors, patterns of health care utilisation, and communication with health professionals (Olsen et al. 2023). These mechanisms are embedded in meso-level social contexts—for example, health literacy is shaped by family and schooling, risk factors by peer and workplace environments, and healthcare utilisation by accessibility and affordability of services. This gives rise to causal pathways in which social structures influence exposures, vulnerabilities, and resources, such as higher occupational carcinogen exposure or clustering of smoking in lower SEP groups. Empirical studies explicitly examining these mechanisms in Germany remain scarce, partly due to limited availability of suitable individual-level data.

## Data availability and evidence in Germany

Despite the increasing relevance of social inequalities in cancer research, the availability of individual-level socioeconomic data that can be linked to cancer outcomes remains highly limited in Germany. A key structural barrier is the absence of a universal personal identification number that could enable deterministic data linkage across administrative, healthcare, and social data sources. In addition, Germany’s data protection regulations and their cautious interpretation (Intemann et al. 2023), combined with limited infrastructural and institutional capacities for secure linkage procedures, has substantially restricted the secondary use of data for epidemiological research. As a result, individual-level analyses of SEP in relation to cancer are rare, and most studies rely either on selected cohort data or on area-based deprivation measures which are too often also used as proxies for individual SEP.

Germany has implemented a nationwide cancer registration for adults. It is organised at the federal level, resulting in fifteen different state cancer registries using the same data standard (Bundesministerium für Gesundheit 2021). These registries have information on diagnosis, treatment, complications and follow-up, and are thus a major source for cancer care research in Germany. However, regarding SEP, only limited information such as place of residence and insurance status is available, which complicates any deeper analysis of the interactions between SEP and cancer.

Individual-level information on SEP in Germany has primarily been derived from cohort studies such as the Socio-Economic Panel (SOEP). Although this dynamic cohort study was implemented in the 1980s and comprises more than 20,000 German households, information may be biased, as information is self-reported and participants were likely healthier than the general population (Hernandez and Schlander 2021). Moreover, cancer-specific information is limited and often relies on self-reported diagnoses (and linkage with cancer registry data has not yet been performed), resulting in only few projects using the SOEP for investigating social inequalities in cancer. Another source for SEP data is the German National Cohort (NAKO), Germany's largest long-term study with over 200,000 participants (Peters et al. 2022). For NAKO, cancer registry linkage is done, the linkage however relies on participants’ specific consent, and the cohort has not yet reached maturity in terms of accumulation of cancer occurrences.

Another potential source of individual SEP data is statutory health insurance (SHI) claims data, which cover nearly 90% of the German population. These data include information on education, occupation and employment status, income-related contribution groups, or unemployment, and researchers have recently started to use SHI data more commonly to analyse social gradients in cancer incidence and survival (e.g. Tetzlaff et al. 2022). However, claims data have important limitations: (1) persons with private health insurance—including many civil servants, self-employed individuals, and high-income earners—are not covered, leading to systematic underrepresentation of higher SEP groups; (2) SEP indicators are often incomplete, not updated in a standardised manner or inconsistently recorded; and (3) detailed tumour characteristics and validated cancer diagnoses are not available and no routine linkages with cancer registry data is performed due to the lack of a lifelong personal identifier across different data sources in Germany. Nevertheless, when used with caution and in combination with other data sources, claims data can help to partially bridge existing data gaps (Tetzlaff et al. 2022).

Given these constraints, most German studies on social inequalities in cancer rely on area-based measures of socioeconomic deprivation. The most widely used index is the German Index of Socioeconomic Deprivation (GISD). The GISD is available nationwide at the district (German “Kreis/kreisfreie Stadt” with an average population of 207,398 persons; min = 34,193 and max = 3,669,491 persons) and municipality level (German “Gemeinde” with an average population of 7701; min = 10 and max = 3,669,491 persons) and is based on the core domains of education, employment, and income (Michalski et al. 2022). An alternative measure is the German Index of Multiple Deprivation (GIMD), which incorporates further domains such as municipal revenue, environment, security, and social capital (Maier 2017). While these indices enable nationwide small-area analyses, they are inherently limited by their ecological nature. Socioeconomic heterogeneity within municipalities or districts may be substantial, especially in large cities such as Berlin or Hamburg, and individual-level inferences are prone to ecological fallacy. Moreover, key dimensions of SEP, such as long-term unemployment, precarious employment, housing conditions, or detailed educational attainment, are only partially captured or entirely absent due to limitations in available administrative data at small spatial scales. Consequently, there is a need to go beyond area-based indices and assess socioeconomic factors and their intersections at the individual level.

Despite substantial data limitations, a growing body of German research consistently demonstrates social inequalities along nearly all stages of the cancer continuum. However, the available evidence still represents only the “tip of the iceberg”, as many relevant dimensions and mechanisms remain insufficiently studied. Thus, the following list of evidence may be interpreted with caution and may be an underestimation of the full impact of SEP regarding cancer, cancer patients and survivors.

*Behavioural risk factors*: In Germany, individual data on cancer-related behavioural risk factors—such as smoking, alcohol use, physical inactivity, and overweight—are more readily available than individual-level socioeconomic data on cancer incidence or mortality. National surveys, for example by the Robert Koch Institute (GEDA, DEGS, KiGGS) or the SOEP provide repeated individual-level information on health behaviours and SEP, enabling analyses of social inequalities and long-term trends in health behaviours (Starker et al. 2025). Analyses consistently demonstrate pronounced inverse gradients by education and income for smoking and obesity (Starker et al. 2025), whereas inequalities in alcohol consumption are more heterogeneous and partly reversed depending on drinking patterns and SEP indicators (Richter et al. 2025). These data form a solid basis for prevention strategies aimed at reducing socioeconomic inequalities in cancer risk.

*Cancer screening*: In Germany, several cancer screening programmes are available and covered by both statutory and private health insurances, with some of them being organised (mammography, colorectal and cervical cancer screening), whereas others are largely opportunistic, such as skin cancer and prostate cancer screening (Gemeinsamer Bundesausschuss 2026, Gemeinsamer Bundesausschuss 2025). Lower participation in cancer screening is consistently observed among individuals with lower SEP and in socioeconomically deprived areas. This applies to mammography (Buschmann et al. 2025), cervical cancer screening, and other preventive services (Pedrós Barnils et al. 2024; Gymah Gyamfi 2024; Kubat et al. 2025).

*Cancer incidence*: Total cancer incidence is higher in socioeconomically deprived areas (Hoebel et al. 2018; Jansen et al. 2023) and among individuals with lower income based on statutory health insurance data (Tetzlaff et al. 2022). The magnitude and direction of socioeconomic inequalities vary substantially by cancer site. The strongest adverse gradients are observed for lung cancer, largely reflecting socially patterned smoking behaviour (Tetzlaff et al. 2022, 2025; Jansen et al. 2023; Brinkwirth et al. 2025). In contrast, the observed incidence of breast cancer, melanoma, and ovarian cancer is higher among higher SEP groups, partly due to differential exposure profiles and higher participation in early detection and screening (Hoebel et al. 2018). In these cancers, higher survival must be interpreted cautiously due to the contribution of overdiagnosis. A multi-level study from Germany indicates that area-level socioeconomic deprivation is linked to the incidence of certain cancers beyond individual-level SEP indicators (Brinkwirth et al. 2025).

*Cancer diagnosis*: At the time of diagnosis, patients from deprived areas and individuals with lower SEP are more likely to present with advanced tumour stage or larger tumour size, indicating delayed detection and potential barriers to timely healthcare access (Rosenbaum et al. 2024; Jaehn et al. 2022).

*Mortality and survival*: Cancer mortality is higher in socioeconomically deprived regions (Tetzlaff et al. 2023). Survival is consistently lower among patients living in deprived areas (Finke et al. 2021) and among individuals with lower SEP (Singer et al. 2017). Analyses based on data from the German Childhood Cancer Registry have found little evidence of socioeconomic inequalities in childhood cancer survival in Germany. One study analysed self-reported individual-level parental socioeconomic status among children diagnosed with leukaemia in the mid-1990s, and the other examined area-based socioeconomic deprivation across all childhood cancer types between 1991 and 2016 (Erdmann et al. 2014; Wellbrock et al. 2025).

*Survivorship*: Socioeconomic inequalities extend into survivorship. Patients with lower education or SEP are less likely to return to work (Breidenbach et al. 2024) and report poorer quality of life and life satisfaction (Safieddine et al. 2025) (Burgmann et al. 2025). Higher levels of anxiety, mental disorders, and fewer perceived positive life changes after cancer have also been documented among socioeconomically disadvantaged survivors (Ernst et al. 2022; Springer et al. 2025). Importantly, cancer itself may act as a driver of socioeconomic disadvantage. Evidence from Germany indicates increased risks of income loss, employment disruption, and long-term financial hardship following a cancer diagnosis, particularly among individuals with lower pre-treatment SEP (Sibert et al. 2025).

## Barriers, challenges and opportunities

Cancer registries are the primary source of clinical and epidemiological data on cancer in Germany. However, they do not routinely collect individual-level SEP information, and, due to the absence of a nationwide lifelong personal identifier used across data sources in Germany and data privacy regulations, linking SEP data to registry records is challenging and rarely done. As a result, researchers often rely on area-based deprivation indices such as the GISD or GIMD (Michalski et al. 2022; Maier and Schwettmann 2018). While these indices provide a broad overview of socioeconomic conditions, they may not accurately capture individual SEP and often omit important dimensions such as long-term unemployment, housing quality, or detailed educational attainment.

Methodological challenges further complicate analyses: the Modifiable Area Unit Problem (Fotheringham and Wong 1991) can obscure intra-regional inequalities, and mixed-area indicators may not fully reflect individual exposures. Additionally, the limited availability of data at finer spatial scales reduces the sensitivity of these measures. Establishing causal relationships between SEP and cancer outcomes remains difficult due to intermediate factors such as healthcare access, behavioural influences, and systemic inequities. Longitudinal, patient-level data are therefore essential to better understand these pathways, including individual as well as contextual information, and to develop targeted interventions.

Beyond technical and methodological issues, a lack of clinical, societal, and political awareness significantly limits progress. Many stakeholders are unaware of how social inequalities shape cancer incidence, diagnosis, treatment, and outcome. This lack of awareness undermines prioritisation efforts for research and policy initiatives aimed at addressing these inequities.

Recent legislative efforts offer pathways to mitigate these barriers. Reforms such as the Research Data Act (German: Forschungsdatengesetz), the Register Act (German: Registergesetz), and the Health Data Use Act (German: Gesundheitsdatennutzungsgesetz) aim to improve data linkage and access to registry and administrative data while upholding rigorous data privacy standards (Schmitt et al. 2023). By facilitating the creation of centralised registries and supporting data sharing through initiatives like the European Health Data Space, these reforms promise to enhance the availability of SEP data and enable more sophisticated analyses (European Commission 2025).

## Patients perspective and ethical considerations and demands

From an ethical standpoint, several aspects warrant consideration, particularly considering the restricted availability of data in Germany. Some colleagues contend that there is a moral responsibility to make health care data generated within the public health system accessible for research that may benefit society as a whole (Ballantyne and Schaefer 2020, 2018; Jungkunz et al. 2024). Such healthcare data shall be considered as a common good, not private property, since it carries the potential of an enormous public benefit, and its use should not be exclusive (Hummel et al. 2021). On the other hand, health data is highly sensitive and harbours the risk of misuse potentially leading to considerable harm for the individual. However, both ethical considerations can be reconciled by allowing the release of health data for academic research purposes, provided that sufficient data security and privacy standards are implemented (Ballantyne and Schaefer 2018).

When considering patient preferences, a close examination of their willingness to share their healthcare data for research purposes is telling. Although research has shown that there is only limited willingness to share data in specific fields, e.g., genomic data, without a specified research purpose (Voigt et al. 2020), this does not hold true for oncology. A survey from Köngeter et al. with over 800 participants has shown a high willingness (97%) among cancer patients to share their healthcare data for research purposes. Most participants even refused to be asked for their consent in every specific case again. However, they demanded strong privacy standards for their data (Köngeter et al. 2022). Additionally, delays or limitations in epidemiological research due to restricted data access may result in avoidable harm to populations, which ethically probably outweighs the rare risk of data misuse when appropriate safeguards are in place.

Therefore, from an ethical as well as the patients’ perspective there is a strong support to make data available for research purposes if high privacy standards are ensured.

## An international comparison

Understanding Germany’s challenges in addressing social inequalities in cancer benefits from an international perspective. Comparing international approaches reveals potential solutions and highlights gaps in Germany, clarifying interventions at macro, meso, and micro levels. Denmark provides a compelling case study. As a country considered to belong to the most egalitarian worldwide, with a extensive welfare system and strong social justice policies, illustrates both the opportunities and limits of addressing cancer-related social inequalities. Comparing Germany and Denmark highlights differences in healthcare structure, policy priorities, and data availability, suggesting actionable steps Germany could take to improve cancer equity.

Denmark’s healthcare system provides universal access: primary and secondary care costs are publicly covered, while dental, allied health, and some medications require partial out-of-pocket payments. This ensures broad access to cancer care, including among the EU’s highest per capita numbers of radiation centres (OECD 2023). Despite this, Denmark still faces significant socioeconomic inequalities in cancer prevention, care, and outcomes. These inequalities are well documented, e.g. in smoking rates (Jensen et al. 2023), obesity prevalence (Rasmussen et al. 2020), and cervical cancer screening, where lower-income or less-educated individuals fare worse (Tabatabai et al. 2023). Nevertheless, Denmark’s robust registry data and its explicit focus on equity in national cancer plans allow for targeted interventions and data-driven policies.

Denmark’s approach benefits from a comprehensive population-based registry infrastructure covering many different topics. The Danish Cancer Registry, among the world’s most comprehensive, has recorded detailed patient data since 1943. Crucially, based on a unique personal identification number that is used in all national registries, individual-level data can be linked across national registries (Schmidt et al. 2014; Pedersen 2011), including education, income, and employment at individual and small-area levels. Danish law permits research use if data are pseudonymised, securely stored, and individuals cannot be identified in publications. This integration allows researchers to explore nuanced relationships between SEP and cancer outcomes (Levinsen et al. 2023). Linking individual-level data allows analysis of social determinants and cancer, providing a comprehensive view of inequalities to inform targeted interventions (Hjorth et al. 2025).

Germany lacks comparable SEP data integration and linkage, limiting scientific evidence. This hinders accurate analysis of social determinants, forcing reliance on area-based deprivation indices. Germany’s fragmented healthcare system and mix of public/private insurance further complicate cohesive strategies and perpetuate disparities.

Documentation of social inequalities by epidemiologists via Danish registries led National Health Authorities to continuously monitor social inequalities in annual Cancer Registry reports. Further, support from large public and private research foundations has led to a development of research from describing SEP impacts to developing interventions to reduce cancer inequalities. Denmark’s National Cancer Plan IV prioritised reducing social inequalities across the entire cancer continuum (Levinsen et al. 2023). Plan V continues to do so, promoting stratified treatment, shared decision-making, prehabilitation, and expanded palliative care to minimize disparities (Ministry for the Interior and Health of Denmark 2025). Germany’s National Cancer Plan, however, lacks a similar focus, reflecting a broader neglect of socioeconomic inequalities in health policy. Limited collaboration among researchers, oncologists and other healthcare providers, and policymakers across different sectors further stymies progress.

Denmark shows the value of comprehensive data, robust data infrastructure, political commitment, and cross-sector collaboration in addressing health inequities. For instance, Denmark has reduced social gradients in breast and colorectal cancer screening rates, demonstrating the effectiveness of targeted interventions. However, persistent challenges, such as higher cancer incidence and mortality rates compared to EU averages, underscore the importance of sustained efforts and adaptable strategies.

Germany can learn from Denmark by integrating or linking individual SEP data into cancer registries, fostering intersectoral collaboration, and embedding equity considerations into national health policy. Targeted, data-informed interventions could reduce disparities and improve equity. An international comparison not only contextualises Germany’s challenges but also illustrates a path forward, where systemic reform and prioritisation of equity can drive meaningful change.

## Call for action

Achieving equity in cancer prevention, diagnosis, treatment, and survivorship in Germany requires urgent and coordinated action. The current gaps in scientific evidence, data availability, awareness, and policy integration call for a comprehensive strategy that addresses both structural barriers and opportunities for improvement.

First, improving the availability and quality of (official) SEP data and possibilities for linkage with cancer data must be prioritised. Reliable, individual-level data that can be linked to cancer registries are essential to understand the full extent of social inequalities and to design targeted and equitable interventions. Standardisation of SEP measures across studies and institutions would increase comparability and reproducibility of evidence, thereby strengthening its impact on clinical and policy decisions. These measures should then be supplemented by indices measuring socioeconomic and multiple deprivation at area level to shed further light on the relevant factors on the meso and macro level.

Second, it is critical to identify vulnerable population groups and address their specific needs. A key outcome of this workshop was that there were no targeted interventions available aimed at reducing social inequality along the cancer continuum. Cancer patients from lower SEP groups often face barriers in screening, diagnostic and treatment access, and survivorship support. Tailored interventions—such as screening invitations, outreach programmes, culturally sensitive health communication, and targeted financial support—are needed to ensure equitable participation along the cancer continuum. Strong patient and community involvement will be needed to design appropriate interventions.

Third, equity considerations must be embedded in Germany’s National Cancer Plan, with equity explicitly stated as a key objective. To date, socioeconomic inequalities as described in German research have received little explicit attention in national cancer strategies. By making SEP an integral part of cancer policy by formulating measurable objectives and consequently monitoring their achievement, Germany would align with international best practices and take meaningful steps towards reducing avoidable inequalities.

Finally, sustained financial and institutional commitment is required. Research funding must support the linkage, collection, and analysis of SEP-related data, while health systems must be equipped to translate evidence into practice. Investments in digital health infrastructure, secure data linkage, and interdisciplinary research will be crucial to ensure long-term impact.

Without decisive action, social inequalities in cancer risk, care and outcomes will likely continue to widen. Policymakers, clinicians, researchers, and patient representatives must now work together to advance an equity agenda for cancer care.

## Conclusion

Cancer is not only a disease of cells and organs but also a mirror of social structures. Addressing inequalities in the cancer continuum can serve as a blueprint for tackling health inequalities more broadly. Insights from cancer research may apply to other chronic diseases with similar socioeconomic gradients, including cardiovascular, respiratory, musculoskeletal, and mental health conditions (Braveman and Gottlieb 2014). General health influences cancer outcomes, as comorbidities affect access to optimal treatment. Therefore, a comprehensive health-in-all-policies approach is needed to address structural causes of inequalities, and cancer strategies should align with nationwide efforts to reduce health disparities.

Promoting equity in cancer can also contribute to a more efficient allocation of healthcare resources and unlock large potential by lowering the burden of advanced disease stages, improving survival rates, and enhancing quality of life, ultimately benefitting society as a whole. Furthermore, it is crucial to highlight not only the underutilisation of cancer care among individuals with lower SEP but also its partial overuse among those with higher SEP (Vaccarella and Vineis 2025). A fairer distribution of healthcare opportunities is not only ethically desirable but also economically rational.

Moreover, the ongoing shift towards personalised medicine should not overlook the dimension of SEP and social inequality. Precision oncology increasingly considers molecular and genetic heterogeneity; however, without addressing social heterogeneity, personalised medicine risks reinforcing rather than reducing inequalities. For example, novel targeted therapies or genomic testing may be less accessible to patients from lower socioeconomic groups due to limited health literacy, financial barriers, or unequal access to specialised cancer centres, resulting in differential uptake of cutting-edge treatments. SEP must therefore be integrated into personalised approaches—both in research and in clinical care—to ensure that innovations in oncology can reach all patients, not only the privileged few.

Ultimately, tackling socioeconomic inequalities in cancer requires a paradigm shift: from seeing them as unfortunate side-effects of social structures to recognising them as unjust, avoidable, and correctable. By embracing this perspective, Germany has the opportunity to take a leading role in advancing equity in cancer prevention, control, care and beyond.

## Data Availability

Not applicable.
